# Hypocapnic cerebral hypoperfusion: A biomarker of orthostatic intolerance

**DOI:** 10.1371/journal.pone.0204419

**Published:** 2018-09-26

**Authors:** Peter Novak

**Affiliations:** Department of Neurology, Brigham and Women’s Faulkner Hospital, Boston, MA, United States of America; University of PECS Medical School, HUNGARY

## Abstract

The objective of the study was to identify markers of hypocapnic cerebral hypoperfusion (HYCH) in patients with orthostatic intolerance (OI) without tachycardia and without orthostatic hypotension. This single center, retrospective study included OI patients referred for autonomic evaluation with the 10 min tilt test. Heart rate, end-tidal CO_2_ (ET-CO_2_), blood pressure, and cerebral blood flow velocity (CBFv) from middle cerebral artery were monitored. HYCH was defined by: (1) Symptoms of OI; (2) Orthostatic hypocapnia (low ET-CO_2_); (3) Abnormal decline in orthostatic CBFv due to hypocapnia; 4) Absence of tachycardia, orthostatic hypotension, or other causes of low CBFv or hypocapnia. Sixteen subjects met HYCH criteria (15/1 women/men, age 38.5±8.0 years) and were matched by age and gender to postural tachycardia patients (POTS, n = 16) and healthy controls (n = 16). During the tilt, CBFv decreased more in HYCH (-22.4±7.7%, p<0.0001) and POTS (-19.0±10.3%, p<0.0001) compared to controls (-3.0±5.0%). Orthostatic ET-CO_2_ was lower in HYCH (26.4±4.2 (mmHg), p<0.0001) and POTS (28.6±4.3, p<0.0001) compared to controls (36.9 ± 2.1 mmHg). Orthostatic heart rate was normal in HYCH (89.0±10.9 (BPM), p<0.08) and controls (80.8 ±11.2), but was higher in POTS (123.7±11.2, p<0.0001). Blood pressure was normal and similar in all groups. It is concluded that both HYCH and POTS patients have comparable decrease in CBFv which is due to vasoconstrictive effect of hypocapnia. Blood flow velocity monitoring can provide an objective biomarker for HYCH in OI patients without tachycardia.

## Introduction

Orthostatic intolerance (OI) represents important health problem and remains poorly understood. Typical symptoms include lightheadedness, dizziness, headache, nausea, fatigue and shortness of breath which are exacerbated during upright position and relieved by recumbency [1]. OI symptoms reflect cerebral hypoperfusion and sympathetic overactivity [2]. In majority of OI patients, orthostatic symptoms occur without orthostatic hypotension [1]. Subjects with OI and orthostatic hypotension may have more widespread autonomic failure, as seen in a progressive neurodegenerative disorders or diabetic or nondiabetic autonomic neuropathies. In the following, the OI will refer to OI without orthostatic hypotension.

Postural tachycardia syndrome (POTS), one of the common form of orthostatic intolerance (OI) is characterized by combination of OI symptoms and postural tachycardia. POTS is the leading cause of disability among women of childbearing age [1]. OI patients that do not meet POTS criteria because of lack of the heart rate criterion are not well defined [1,3,4]. The true prevalence of OI is unknown, but considering that it is estimated that POTS—a subset of OI—affects 500,000–3,000,000 people in the US [5], the occurrence of OI may be at least twice as much or more. Therefore, there is an urgent need for accurate diagnostic criteria of OI.

Cerebral hypoperfusion in OI can be related to hypocapnia which causes cerebral vasoconstriction and reduces cerebral blood flow. This mechanism, e.g. hypocapnic cerebral hypoperfusion, was described in POTS [2]. The main hypothesis is that the cerebral hypoperfusion due to hypocapnia-induced vasoconstriction s and underlying mechanisms of OI symptoms in patients with history of OI without tachycardia. Cerebral blood flow velocity using transcranial Doppler has been used as a proxy for cerebral blood flow measurements [6]. This study compared the OI patients without tachycardia with POTS and healthy controls using autonomic testing with end tidal CO_2_ and cerebral blood flow monitoring.

## Material and methods

This retrospective, single-center study included consecutive patients who underwent autonomic testing between 2016 and 2017 at the Brigham and Women’s Faulkner Hospital Autonomic laboratory for evaluation of orthostatic intolerance. Patient’s electronic records were reviewed for details about past medical history, laboratory evaluations, including blood work and, as well as the use of medication. Healthy controls data were referenced from our Autonomic database at the University of Massachusetts [7].

### Standard protocol approvals, registrations, and patient consents

The study was approved by the Institutional Review Board of the Brigham and Women’s Hospital, Harvard University, as a minimal risk study and the consent form signature was waived.

### Clinical definitions

OI was defined as the presence of symptoms of cerebral hypoperfusion with standing and relieve of symptoms by recumbency [1]. The characteristic OI symptoms include lightheadedness or dizziness, palpitations, heat intolerance, sense of weakness, tremulousness, exacerbation by meals, hyperhidrosis exercise intolerance and shortness of breath [1]. Cerebral blood flow depends principally on systemic blood pressure and end tidal CO_2_.

HYCH was defined by the following inclusion criteria: 1) OI symptoms; 2) orthostatic hypocapnia during the tilt test defined as end tidal CO_2_ < 30 mmHg; 2) reduced orthostatic cerebral blood flow velocity (CBFv) that can be explained by a decline in end tidal CO_2._ Functioning cerebral autoregulation keeps the orthostatic cerebral blood flow stable within the autoregulatory range which is 60–150 mmHg of mean blood pressure[8]_._ With normal cerebral autoregulation and in the absence of orthostatic hypotension, changes in CBFv can be attributed to variations in end tidal CO_2_. Hypocapnia induces cerebral vasoconstriction and reduces CBFv. A decrease in end tidal CO_2_ by 1 mmHg decreases CBFv by about 3% [8]; POTS was defined as a symptomatic increment of heart rate ≥ 30 BPM and exceeding 1209 BPM [1]. Reduced orthostatic CBFv without decline in end tidal CO_2_ would be consistent with orthostatic cerebral hypoperfusion syndrome (OCHOS) [9].

The HYCH and POTS subjects were matched with a control group by age, gender, and body mass index. All controls were asymptomatic and had normal response to tilt in heart rate, blood pressure, CBFv and respiratory variables.

Exclusion criteria for both HYCH and POTS group were: (1) presence of orthostatic hypotension; (2) presence of arrhythmia, or bradycardia (HR < 50 BPM) during supine or tilt test; (3) inability to complete the 10 min tilt test for any reason including syncope; (4) any structural abnormality on brain CT or MRI imaging that could cause significant hemodynamic deficit;(5) any other cause of abnormal intracranial velocities, including cerebrovascular accident affecting large vessels, abnormal hematocrit; (6) the use of medication that affect autonomic functions, including vasoactive medication (vasodilators or vasoconstrictors) or medication that can cause orthostatic tachycardia; (7) any pulmonary, cardiovascular or systemic disorder that can cause hypocapnia including chronic fatigue syndrome and fibromyalgia [10]; (9) Hyperadrenergic state defined as standing plasma norepinephrine ≥ 600 pg/ml. This will exclude the hyperadrenergic form of POTS that may have different etiology than neuropathic POTS [3]; (10) Patients with unavailable or incomplete medical records were also excluded.

### Autonomic tests

All testing was performed following established standards as previously described [11]. Cardiovascular reflex tests included deep breathing, the Valsalva maneuver, and the tilt test. Patients were tilted at 70° for 10 min or more, after 10 min of supine rest. Recorded signals included electrocardiogram, blood pressure, respiratory movements and rate end tidal CO_2_, CBFv in the middle cerebral artery using transcranial Doppler. Baseline supine blood pressure was obtained intermittently using an automated oscillometric blood pressure monitor Welch Allyn CVSM 6400 Monitor (Skaneateles Falls, NY) from arm and continuously using Finometer^®^ (Finapress Medical Systems, Amsterdam, Netherlands) from the third finger. During the tilt test, blood pressure was obtained every minute using the CVSM 6400 Monitor and continuously using Finometer^®^. Finometer-based blood pressure was calibrated with the CVSM 6400 based blood pressure. End tidal CO_2_ was obtained using Nonin Respsense Capnograph (Nonin Medical Inc. Plymouth, MN). A pulse oximeter (part of Welch Allyn monitor) was used to monitor the oxygen saturation usually from the left middle finger throughout the testing.

Right middle cerebral artery was insonated at the temporal acoustic window with a 2 Mhz probe at depth between 45 and 65 mm using a Transacranial Dopper systemMultiDop T (Multigon, New York). Transducer was attached with a head frame with a three-dimensional positioner to maintain a tight transducer fixation at the constant depth and angle. Continuous trancranial doppler monitoring was performed during the supine baseline period and during the tilt. Signals were recorded using PowerLab 16/35 data acquisition system with LabChart 8 software (ADInstruments Inc., Colorado Springs, CO, USA) and sampled at 400 Hz.

Normative data for CBFv at both supine and tilt test depend on age, gender and the duration of tilt. In healthy subjects, CBFv remains either unchanged or there can be a mild drop in CBFv during the tilt. The criteria for normal drop of the mean CBFv during tilt test are equal to 90/% (1st minute), 89% (5th minute), and 85% (10th minute) of the tilt where baseline is equal to 100% [12].

The heart rate, blood pressure, CBFv and end tidal CO_2_ were analyzed at the supine baseline immediately before the tilt, and at the 1st, 5th, and 10th minute of tilt. Cerebral vascular resistance were also calculated as mean blood pressure/mean CBFv[2].

All subjects were observed for presence of orthostatic symptoms during tilt test. All subjects were assessed for the presence of sensory complaints that included self-reported the Neuropathy Total Symptom Score-6 (NTSS6) [13]. The Survey of Autonomic Symptoms [14] was used to assess the frequency and severity of autonomic symptoms.

### Statistical analysis

One-way analysis of variance (ANOVA) was used to test differences between the HYCH, POTS and healthy controls at baseline, 1st minute, 5th minute, and 10th minute of tilt. The measurements at these intervals (e.g. 1,5 and 10 minutes of the tilt) were analyzed since previous validation studies showed clinical relevance of these intervals[12,15]. In particular it was shown that measurement at these intervals stratify subjects based on severity of autonomic failure [15] as well as on severity of abnormal cerebral blood flow velocity [15] in subjects with graded dysautonomia including diabetes, autonomic neuropathies, Parkinson’s disease and multiple system atrophy.

If significant, the between pairs comparisons were done using the Student’s test. The initial significance level (α) has been set to 0.05. Since there were four comparisons, the Bonferroni-corrected significance level was adjusted to 0.0125 (obtained by 0.05/4). JMP 12 (Cary, NC, USA) statistical software was used for all statistical analyses. The measurements between the HYCH, POTS and healthy controls at supine baseline, 1st minute, 5th minute, and 10th minute of tilt were also compared using repeated measures MANOVA.

Power analyses: CBFv is the main clinical variable of interest which separates HYCH from controls. Based on our data with 32 subjects (HYCH and controls) we have >99% to detect a difference 14.8 cm/s in mean CBFv at the minute 10 of the tilt with alpha 0.01.

## Results

Sixteen patients (15/1 (women/men), (age (years)) 38.6±8.1, (BMI(kg/m^2^)) 25.3 ± 5.0), satisfied criteria for HYCH. These patients were age- and gender-matched to 16 POTS patients (14/2, 33.2 ± 6.5, 24.1 ± 7.1) and 16 healthy historical controls (13/3, 35.8 ± 7.6, 27.1 ± 3.9).

**Fig 1:**
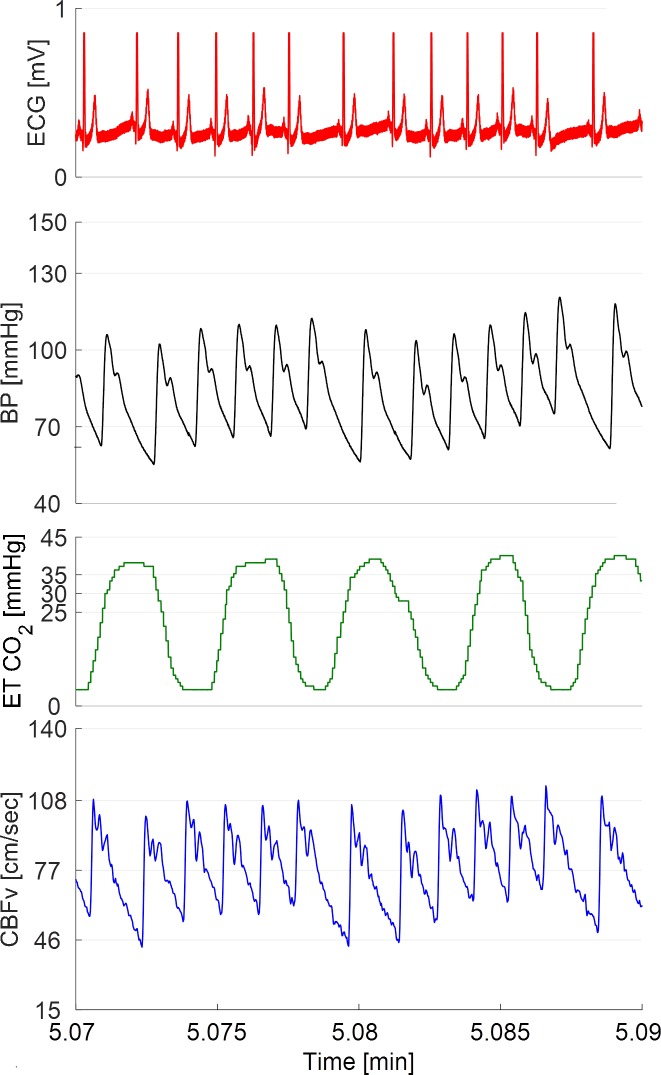
Example of continuous raw data. From top to bottom: electrocardiogram, beat-to-beat blood pressure, end tidal CO_2_ and cerebral blood flow velocity.

**Fig 2:**
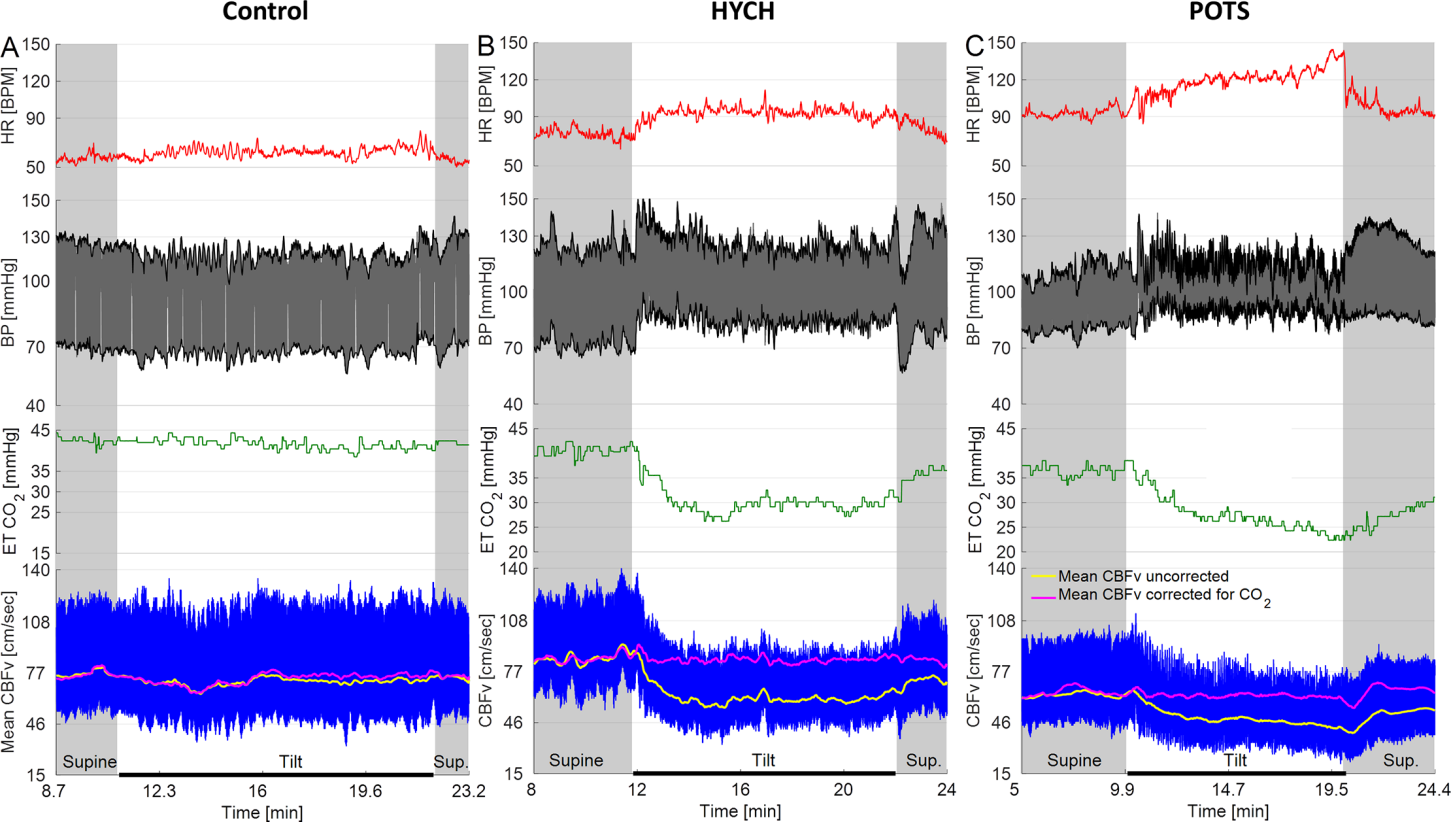
Representative examples of control, HYCH and POTS.

**Fig 3:**
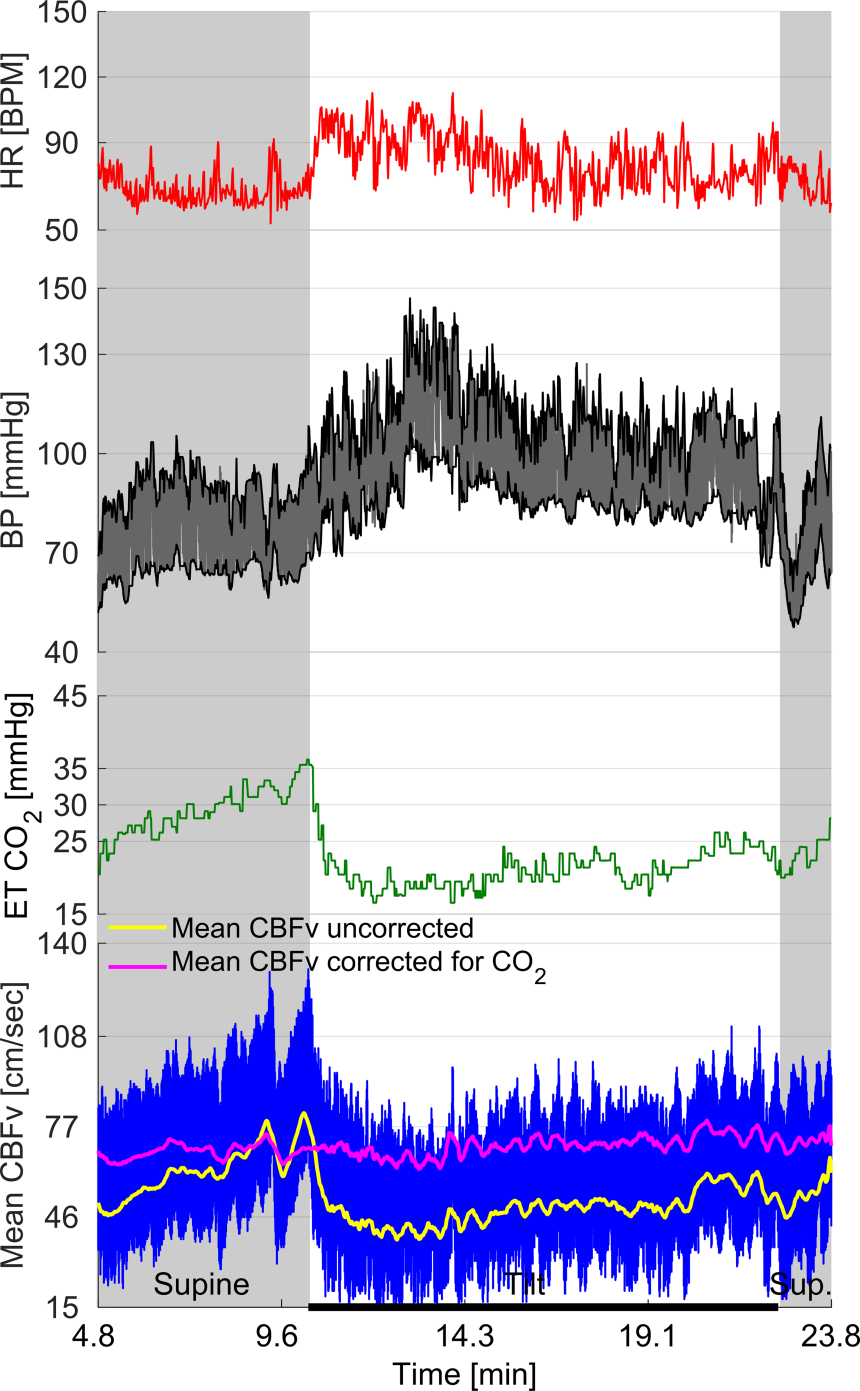
Case of severe HYCH. 21 y/o woman presented with a sudden onset of shortness of breath, chest pain, fatigue and dizziness 2 years ago. Previously highly functioning women is on disability. Heart rate and blood pressure responses to tilt were normal (A). Patient was hypocapnic even at supine with end tidal CO_2_ < 35 mmHg. End tidal CO_2_ declined to 15 mmHg during the tilt which was associated with a drop of mean cerebral blood flow velocity (CBFv) about 50%. Just before the tilt the end tidal CO_2_ increased to 35 mmHg that temporarily normalized CBFv demonstrating that the decline in CBFv was due to hypocapnia.

**Fig 4:**
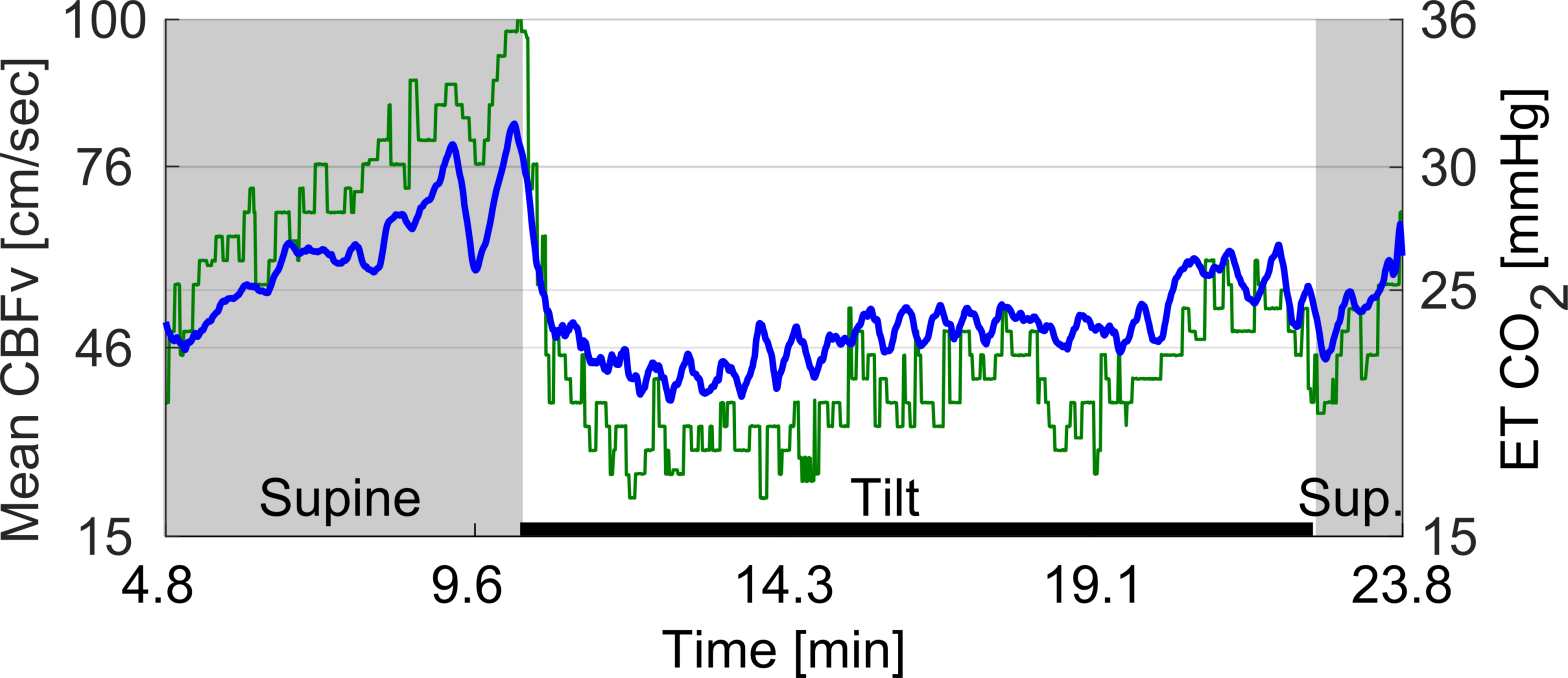
Superimposed end tidal CO_2_ and mean cerebral blood flow velocity from the same patient as in Fig 3.

All subjects with HYCH and POTS had at least one orthostatic symptom during the tilt test while controls were asymptomatic (Table 1). There was no difference between HYCH and POTS in frequency of autonomic symptoms (6.2 ± 2.9 versus 6.1 ± 3.5, p<0.87), autonomic symptoms impact (19.7 ± 10.5 versus 19.2 ± 13.4, p< 0.90) and NTSS6 sensory score (6.2 ± 3.9, versus 5.9 ± 3.8, p<0.82). An example of recorded signals is shown in the Fig 1. Representative examples of a control, HYCH and POTS are in the Fig 2. The HYCH patient had a notable decrease in CBFv and ends tidal CO_2_ with normal heart rate and blood pressure. The POTS patient has a decrease in CBFv, end tidal CO_2_, postural tachycardia and normal blood pressure. Figs 3 and 4 show a severe case of HYCH with profound decrease in CBFv in supine and during the tilt.

**Table 1:** Frequency of symptoms in patients with HYCH and POTS.

Symptom	HYCH	POTS
Lightheadedness	15	14
Dry mouth or dry eyes	11	9
Pale or blue feet	7	10
Feet colder than the rest of body	13	8
Decreased sweating at feet at rest	5	4
Decreased sweating at feet after exercise or during hot weather	5	4
Sweating increased at hands	7	9
Nausea, vomiting or bloating after meal	9	9
Persistent diarrhea	9	8
Persistent constipation	8	8
Leaking of urine	6	5
Difficulties in erection (man)	0	0
Aching pain	14	12
Allodynia	3	2
Burning pain	4	3
Lancinating pain	5	5
Numbness	12	9
Prickling sensation	5	3

### Group comparisons using autonomic testing

#### The supine baseline

Supine end tidal CO_2_ was reduced in HYCH (33.2 ± 3.3 mmHg, p<0.0002) and POTS (33.6 ± 4.1mmHg, p<0.0007) compared to controls (38.0 ± 2.1 mmHg). There was no difference between HYCH and POTS in end tidal CO_2_. There was no difference at the baseline in all other variables. Supine baseline CBFv (cm/sec) was 65.5 ± 7.6 (controls), 65.9 ± 9.6 (HYCH) and 66.1 ± 8.1 (POTS).

#### Tilt

**Fig 5:**
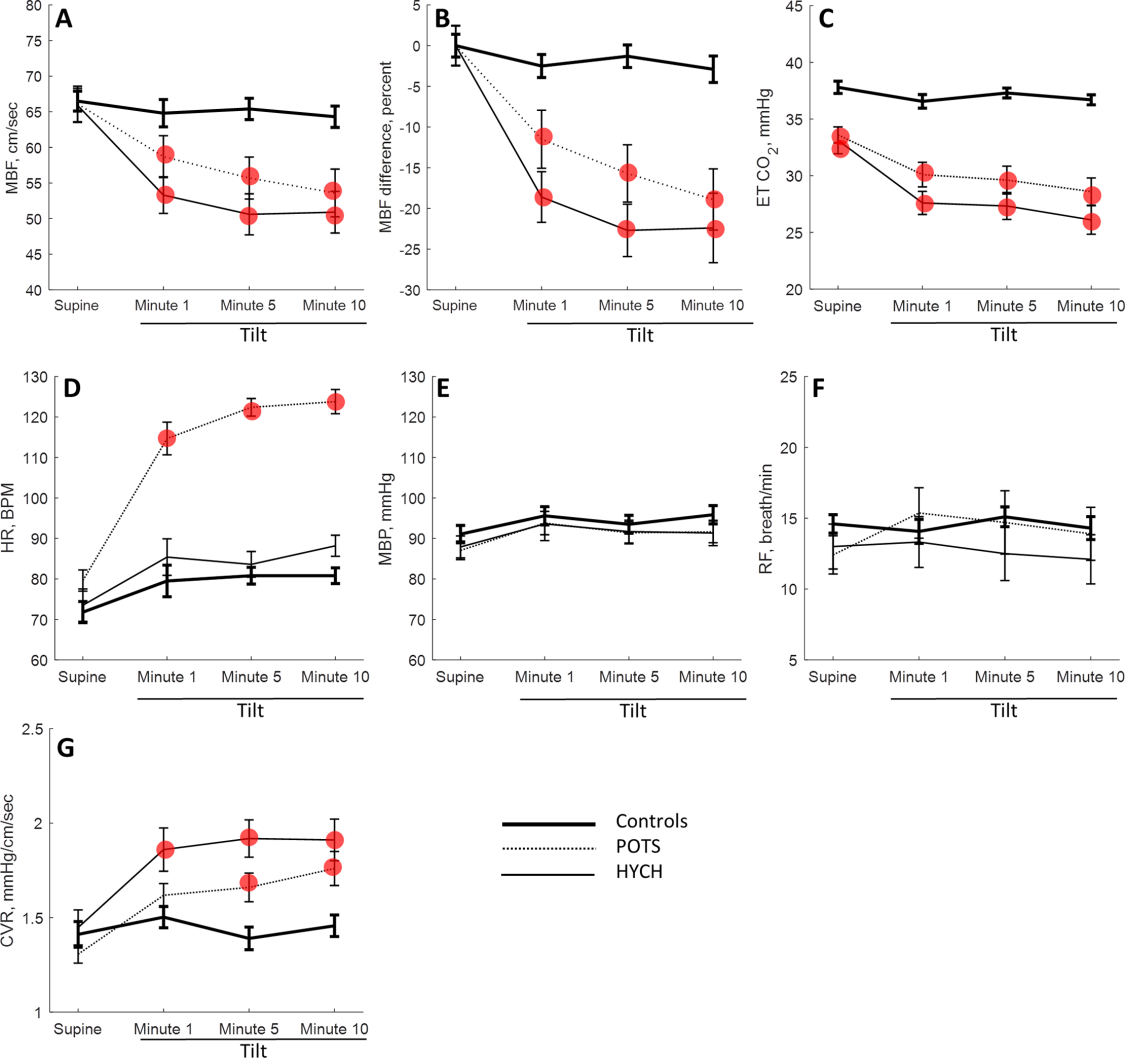
Comparison of HYCH, POTS and controls. The hemodynamic variables were analyzed during supine position and at the minute 1, 5 and 10 of the tilt. Red circle shows significance difference compared to controls. A. CBFv = mean blood flow velocity, B. CBFv difference = percent difference in CBFv compared to supine baseline; C. ET = end tidal CO_2_; D. HR = heart rate, E. CVR = cerebrovascular resistance, F. MBP = mean blood pressure;, G. RF = respiratory frequency.

Mean CBFv declined and the percent CBFv difference from baseline increased during the tilt (the following numbers apply to the 10 the minute of the tilt) in HYCH ((mean) 50.9 ± 6.8 cm/sec, p<0.0001 (the difference from supine baseline) -22.4 ± 7.7%, p<0.0001) and POTS (53.6 ± 9.5 cm/sec, p<0.0001, -19.0 ± 10.3%, p<0.0001) compared to controls (64.3±6.8 cm/sec, -3.0±5.0%, Fig 5). The mean CBFv (p = 0.0008) and percent change (p<0.0001) of CBFv was different among the groups during the tilt (MANOVA). However, CBFv adjusted for end tidal CO_2_ was not different between all groups (p = 0.32). End tidal CO_2_ was reduced in HYCH (26.4 ± 4.2 mmHg, p<0.0001) and POTS (28.6 ±4.3 mmHg, ANOVA p<0.0001, MANOVA p<0.0001) compared to controls (36.9 ± 2.1 mmHg). Heart rate was normal in HYCH (89.0 ± 10.9 (BPM), p<0.08) and controls (80.8 ± 11.2), but higher in POTS (123.7 ± 11.2, p<0.0001). CVR was elevated in HYCH (1.90 ± 0.40, p<0.0012) and POTS (1.75 ±0.32, p<0.01) compared to controls (1.45 ± 0.19). Both disease groups have higher CVR (MANOVA), p = 0.006. End tidal CO_2_ was higher in controls (MANOVA, p<0.001). There was no difference between HYCH and POTS in CBFv and in end tidal CO_2_. Blood pressure and respiratory frequency was normal and similar between the groups. In all subjects, the oxygen saturation was within normal limits throughout the testing (range 96–99%).

## Discussion

This study describes a distinct cohort of OI patients with hypocapnic cerebral hypoperfusion. The main finding is that during the tilt, the mean CBFv decreased 22.4% in HYCH subjects which was similar to POTS (19%), as compared to only 3% in controls. The study is statistically powered enough to interpret the results as being significant. Second finding is that HYCH patients had abnormally low orthostatic end tidal CO_2,_ similarly to POTS patients. However, HYCH patients had a normal heart rate and blood pressure responses to tilt. Blood pressure remained stable during the tilt in all groups, as patients with syncope or orthostatic hypotension were excluded. Therefore, chronic hypocapnia may be an underlying feature of orthostatic intolerance independent of tachycardia.

### Significance and cause of low CBFv

Many symptoms in POTS can be attributed to cerebral hypoperfusion [1,2]. This study suggests that HYCH is also associated with cerebral hypoperfusion and vasoconstriction. Notably, HYCH and POTS patients have similar OI symptoms, have comparable orthostatic decline in CBFv, and are of similar age and gender; therefore it is conceivable that reduced CBFv is also associated with cerebral hypoperfusion in HYCH patients. Reduction in orthostatic CBFv by 19% from the supine baseline period is associated with signs of central nervous system dysfunction in POTS patients [2,16] which is similar to the decline found in HYCH patients observed in this study.

HYCH subjects had abnormally low end tidal CO_2._ and elevated CVR during the tilt, similarly as POTS subjects. Since by definition patients with orthostatic hypotension were excluded, then cerebral vasoconstriction induced by hypocapnia explains the reduction in CBFv in HYCH, a mechanism already observed in POTS [2]. Normally, a decrease of end tidal CO_2_ by 1 mmHg decreases cerebral blood flow by about 3% [8]. HYCH subjects had exacerbated decrease in orthostatic end tidal CO_2_ by 7 mmHg with predicted decrease in CBFv by 21%, that was equal to the CBFv obtained in this study.

### Pathophysiology of hypocapnia

Hypocapnia is frequently encountered in clinical practice [17]. The important question to be answered is the origin of hypocapnia observed in HYCH, e.g. whether it is caused by physiologic mechanisms (secondary hypocapnia) or by psychological factors (primary hypocapnia). The known causes of secondary hypocapnia were excluded in HYCH subjects including pulmonary disorders, hypotension, severe metabolic abnormalities, central nervous system disorders, drugs and pain. It is still possible that there are other physiologic factors which may drive hypocapnia.

The primary hypocapnia, either psychogenic or anxiety related, can be a part of hyperventilation syndrome (HSV). HSV refers to a variety of somatic symptoms which are caused by hyperventilation [18,19]. Patients with HVS has history of recurrent dyspnea, dizziness, or paresthesia typically associated with respiratory alkalosis during hyperventilation.[18] Postural hyperventilation associated with drop in end tidal CO_2_ has been observed in HVS patients [19]. Some patients with HVS and postural hypocapnia had exacerbated tachycardia during standing [19] satisfying current POTS criteria.

Hypocapnic hyperventilation has been previously described in other conditions such as neurally mediated syncope [10,20], chronic fatigue syndrome [10,21] and fibromyalgia [10] which may are often present in the heterogeneous group with OI symptoms. However, these conditions were excluded from our study.

It remains to be established if HYCH group represents a variant of hyperventilation syndrome. The concept of hyperventilation syndrome itself has been questioned because hyperventilation may be a consequence rather than a cause of the HVS disorder [18]. Although hypocapnia and related decrease in cerebral blood flow has been described in POTS [2], its cause even in POTS is still not satisfactory explained. Therefore, it can only be speculated that potential causes of hypocapnia in HYCH, and probably also for POTS, include a baroreceptor driven changes in respiratory drive [22], compensation for metabolic acidosis, orthostatic ventilation-perfusion mismatch, change of responsiveness of respiratory centers in the brain and perhaps other mechanisms related to orthostatic challenge.

### Effect of hypocapnia on the brain

In addition to reduction of cerebral blood flow, hypocapnia associated with respiratory alkalosis may have direct effect of brain functioning. CO_2_ is highly permeable to brain blood barrier and hypocapnia increases cerebral pH [23] which has multiple effects including alterations in neuronal excitability.[24]

### Relationship between HYCH and POTS

HYCH and POTS have similar clinical presentations; in fact more than 80% of patients with HYCH were referred for evaluation of suspected POTS. Historically postural tachycardia has become a major criterion for OI. Therefore many patients without tachycardia remain undiagnosed by the tilt test if the end tidal CO_2_ and CBFv are not measured. Clinical and hemodynamic variables at baseline and during tilt were similar between HYCH and POTS except of absent tachycardia in HYCH. Then the question arises whether both HYCH and POTS represent different diagnostic entities or they represent a spectrum of the same disorder of orthostatic intolerance. POTS is a syndrome of multiple causes. It is possible that there is overlap between HYCH and some forms of POTS. Nevertheless, further studies are necessary to establish link between HYCH and POTS and other disorders of OI.

### Study implication

This study emphasizes the importance of respiration in OI as hypocapnia underlies abnormal CBFv and likely results in cerebral hypoperfusion in OI. Future interventions may be directed to alter the respiratory dynamics perhaps in combination with reconditioning [4] which may improve outcomes of patients with OI.

### Study limitations

This study has number of limitations. The study was retrospective where the diagnostic criteria were applied to already acquired data. A referral bias may affect selection of subjects and, therefore, the studied population may not be representative. Furthermore, the studied population has been narrow as it did not include other disorders of OI. However, the purpose of this study was to exclude disorders with multifactorial causes such as syncope, chronic fatigue syndrome or fibromyalgia in order to demonstrate the link between hypocapnia and low CBFv in well-defined OI population. Other technical aspects may influence the results. The cerebral blood flow was assessed indirectly using CBFv. Transcranial Doppler measures flow velocity instead of blood flow. The velocity is not only proportional to flow but also depends on the diameter of the insonated vessel. The MCA diameter does not change during orthostatic stress[6] and, therefore, CBFv is a good substitute of flow [25]. The study also did not evaluate deconditioning which may play a role in OI [4]. However a previous study found similar rate of deconditioning in OI with (95%) and without (91%) orthostatic tachycardia[4] further strengthening link between OI with and without tachycardia. The volume depletion, which may contribute to OI [3], was not assessed directly. Nevertheless, indirect markers of severe volume depletion such as elevated blood urea nitrogen, elevated serum creatinine, supine tachycardia, diminished skin turgor and dry mucous membranes [26] were absent in all studied groups and most POTS patients do not have reduced plasma volumes [1]. Another shortcoming is the absence of tidal volume in this study, commonly used to evaluate respiratory functions. However, it was shown previously that tidal volume is elevated in POTS with hypocapnia [27]. Tidal volume is a function of end tidal CO_2_ and of respiratory frequency [28], therefore it is likely that HYCH and POTS subject have similar tidal volume since both groups have similar respiratory frequency, end tidal CO_2_, normal oxygen saturation as well as basic demographic characteristics (body mass index, age, gender).

## Conclusion

Decreased of cerebral blood flow and end tidal CO_2_, as described with HYCH, can be an objective biomarker of OI subjects without tachycardia. Therefore, continuous monitoring of cerebral hemodynamic and end tidal CO_2_ provides additional diagnostic value in symptomatic OI patients without tachycardia. The spectrum of orthostatic intolerance is broad and includes several overlapping presentations. A decrease in orthostatic cerebral blood flow may be one of the unifying mechanisms of OI.
